# Preparation of trinucleotide phosphoramidites as synthons for the synthesis of gene libraries

**DOI:** 10.3762/bjoc.14.28

**Published:** 2018-02-13

**Authors:** Ruth Suchsland, Bettina Appel, Sabine Müller

**Affiliations:** 1 Institut für Biochemie, Ernst-Moritz-Arndt-Universität Greifswald, Felix-Hausdorff-Str. 4, D-17489 Greifswald, Germany

**Keywords:** gene library, protein engineering, soluble support, synthesis, trinucleotide

## Abstract

The preparation of protein libraries is a key issue in protein engineering and biotechnology. Such libraries can be prepared by a variety of methods, starting from the respective gene library. The challenge in gene library preparation is to achieve controlled total or partial randomization at any predefined number and position of codons of a given gene, in order to obtain a library with a maximum number of potentially successful candidates. This purpose is best achieved by the usage of trinucleotide synthons for codon-based gene synthesis. We here review the strategies for the preparation of fully protected trinucleotides, emphasizing more recent developments for their synthesis on solid phase and on soluble polymers, and their use as synthons in standard DNA synthesis.

## Introduction

Protein engineering is a highly actual research area with a number of potential applications [1–4]. The construction, adaptation and optimization of proteins can proceed by two major strategies: (i) rational design or (ii) directed evolution. The rational design is based on the introduction of point mutations, insertions or deletions at a defined position of the protein sequence, and requires detailed knowledge of the protein structure and the mechanism of action. On the opposite, directed evolution relies on the selection of a mutant with predefined properties from a random protein library. This strategy is advantageous over the rational design; whenever molecular properties of proteins are investigated that are not yet sufficiently understood, if properties like solvent or temperature stability need to be optimized, or regio-, chemo- or enantioselectivity and substrate specificity shall be changed. Thus, the optimization and variation of proteins, in particular of enzymes, by random mutagenesis and subsequent selection and identification of mutants with improved properties is a favoured method in the field of white biotechnology and biocatalysis, to improve the fitness of enzymes for industrial application [5].

**Figure 1:**
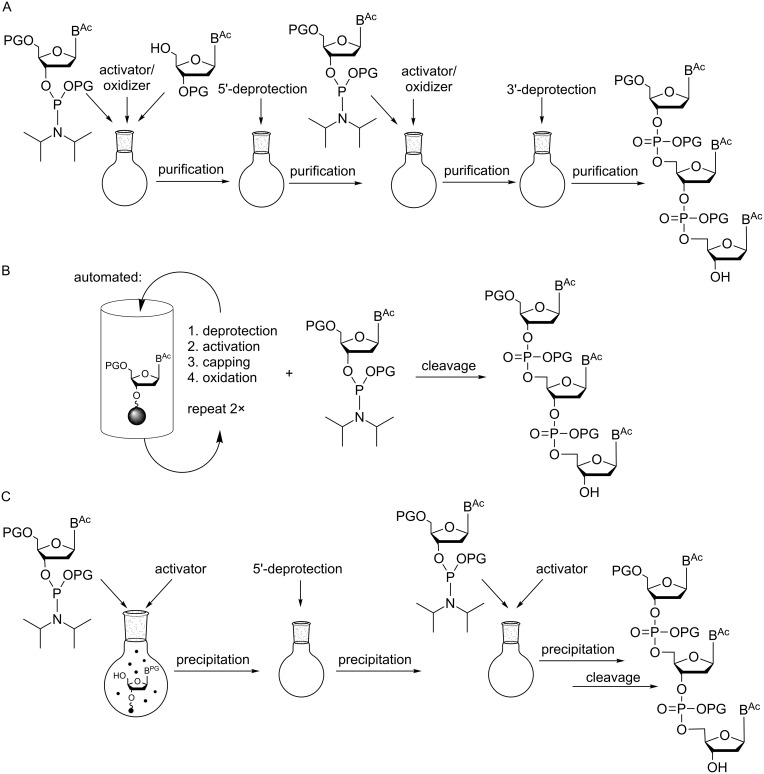
Preparation of fully protected trinucleotides in solution (A), on solid phase (B) and on soluble polymers (C).

In general, directed evolution may be summarized as an iterative two-step process which involves the generation of protein mutant libraries and high throughput screening processes to select for variants with improved traits. Protein mutant libraries are produced from gene libraries, which are generated by random mutagenesis at DNA level. Often polymerase chain raction (PCR)-based methods like error-prone PCR are used for this purpose as well as recombinant methods like DNA shuffling and related strategies [6–7]. One of the major challenges in gene library production is to generate libraries with a high number of promising candidates to enhance the chance of selecting functional protein variants. The methods mentioned above allow the degree and localization of randomization to be adjusted to a certain degree, however, full control over mutagenesis is still rather limited. Oligonucleotide-based methods with a number of sophisticated techniques [8] are advantageous here, as they offer a better possibility to control randomization. The basic principle consists of using chemically synthesized primers of mixed composition for introducing subsets of the 20 canonical amino acids at a defined position of the protein [9]. In the simplest way, a mixture of the four standard nucleotides is used for coupling at each randomized position of the primer in DNA synthesis. For a primer with 9 randomized positions (corresponding to three randomized amino acids in the resulting protein) this would lead to 4^9^ = 262144 sequence variants including stop codons and codons of undesired amino acids, and a bias towards amino acids encoded by multiple codons. Moreover, it is impossible to restrict randomization to a defined subset of amino acids at a desired position. Thus, the result is a rather large library, however, with only a small number of potentially successful candidates. There are strategies to at least partially circumvent this problem, like using NNS instead of NNN codons (with N = A, C, G, T; S = C, G) taking advantage of redundancy of the third nucleotide positions in the majority of codons [10], or using spiked oligonucleotides [11], which are synthesized from solutions of the four nucleotide building blocks, each of those contaminated with a "spiking mix" consisting of equal aliquots of each of the four building blocks [9,12]. The required volume of the spiking mix to achieve a desired amount of nucleotide replacements at a defined position of the oligonucleotide can be calculated, such that library size and degree of randomization can be restricted [13–14]. Nevertheless, although those methods and sophisticated variations of them [14–17] have improved library design and synthesis, full control over randomization is not possible. This can be achieved only by the usage of trinucleotide synthons for codon-based synthesis of a desired primer [18]. Taking the example from above, for a DNA fragment encoding three randomized amino acids, instead of nine nucleotide positions to be randomized, variation of trinucleotides (codons for the 20 amino acids) at only three positions is required. Therefore, the number of possible sequence variants in the gene library decreases from 4^9^ = 262144 to 20^3^ = 8000, if the full set of the 20 amino acids is desired at each of the three randomized positions. The library size can be even further decreased by using subsets of amino acids (e.g., only basic or only acidic amino acids) at the individual positions. Furthermore, stop codons as well as bias to amino acids with codon redundancy are completely prevented. Not at last, the coupling efficiency of individual trinucleotide synthons in chemical DNA synthesis can be considered when preparing the trinucleotide mixture, to ensure that each of the trinucleotides is coupled with identical statistical probability, or alternatively, to adjust the trinucleotide mixture to a desired amino acid distribution at the respective position. Thus, the application of trinucleotide building blocks for the synthesis of gene libraries stands out as facilitating fully controlled total or partial randomization at any predefined number and position of codons of a given gene. Trinucleotide synthons need to be chemically synthesized. Here, the challenge has been to find a suitable set of orthogonal protecting groups that allows the preparation of the trinucleotide, its conversion into a coupling competent building block, and its subsequent use in chemical DNA synthesis. Trinucleotides have been prepared in solution [19], on solid phase [20], and more recently on soluble polymers [21–23] (Figure 1), followed by phosphitylation to be used in standard DNA synthesis.

The preparation of mixed oligonucleotides for random mutagenesis including the strategy of using trinucleotide synthons has been reviewed recently [19,24]. Therefore, herein we will concentrate on more recent developments in trinucleotide synthesis.

## Review

### 1. Preparation of trinucleotides in solution

**Figure 2:**
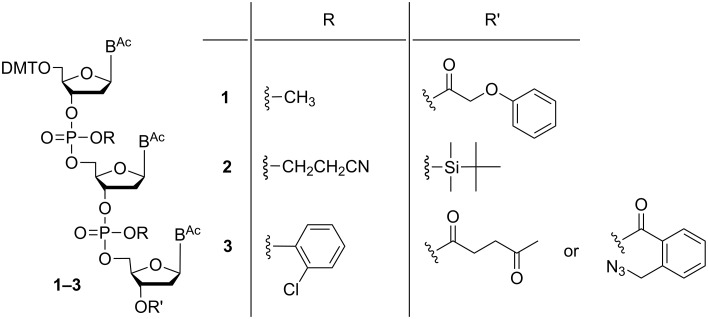
Strategies for trinucleotide synthesis using different pairs of orthogonal groups for protection of the phosphates and the 3'-OH-function.

Over the years, a number of methodologies has been published, varying in the protecting group for the phosphate moiety being methyl [25], ethyl [26], cyanoethyl [27] or *ortho*-chlorophenyl [28–29], and for the 3'-OH-group being phenoxyacetyl [25], dimethoxytrityl (DMTr) [26], *tert*-butyldimethylsilyl (TBDMS) [27,30], levulinoyl [26], or 2-azidomethylbenzoyl [27] (Figure 2), and applying either phosphotriester chemistry [28–29,31–32] or phosphite triester chemistry [25–27,30] in solution.

**Figure 3:**
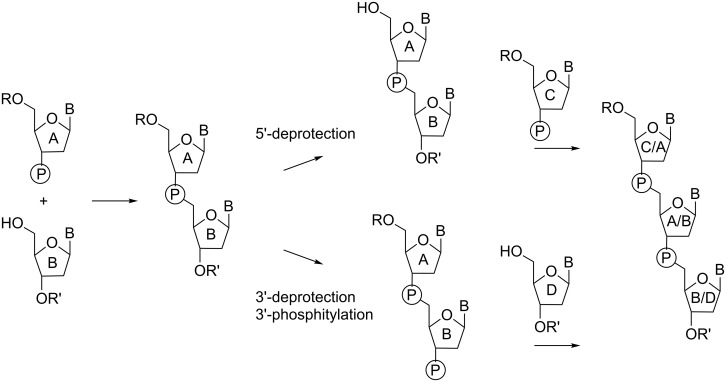
Strategy for the synthesis of nucleotide dimers and extension to the trimer in either 5'- or 3'-direction.

In general, trinucleotides can be assembled through the reaction of two suitably protected monomers to generate a dinucleotide, which then can be extended in either 5'- or 3'-direction (Figure 3).

Surprisingly, only one report has made use of this "economy", first coupling a 5'-*O*-DMTr-protected nucleoside-3'-*ortho*-chlorophenylphosphotriester to a 3'-*O*-levulinoyl-protected monomer. Upon selective removal of either the 5'-*O*-DMTr group or the 3'-*O*-levulinoyl group, the dimer was extended in 5' or 3' direction [26]. All other reports describe strategies, where the dimers are extended unidirectional, either in 5'-direction [25–27,29–30] or 3'-direction [31–32]. A key issue in all these methodologies is that the 5'- or the 3'-O-protecting group is selectively cleaved, whereas all other protecting groups (at the nucleobases, the phosphorous and the 5'- or alternatively 3'-OH group) remain intact.

**Figure 4:**
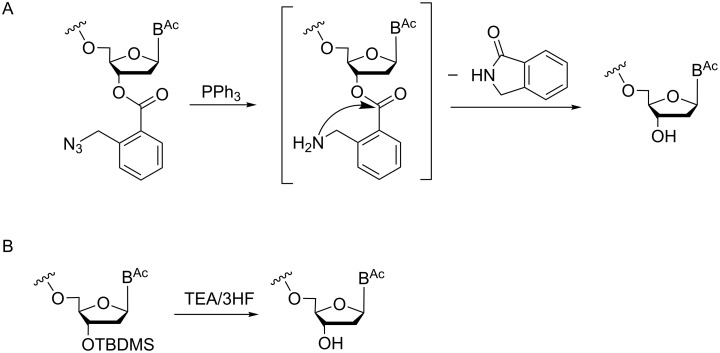
Removal of the 3'-O-protecting group under conditions that leave all other protecting groups at 5'-OH, nucleobases and internucleotide phosphates intact.

Basically, this aim has been achieved, although in particular in earlier reports a number of problems associated with insufficient stability of protecting groups under synthesis conditions, as well as restricted orthogonality have been described, which was mirrored in the sometimes severely limited quality of the trinucleotide synthons and accordingly of the prepared oligonucleotide libraries [14–15,25–26,28,30–31,33]. Among the described procedures the use of *tert*-butyldimethylsilyl [25] and 2-azidomethylbenzoyl groups [29] for 3'-O-protection stands out as being the most successful in terms of high quality trinucleotides. Both protecting groups, under the applied conditions, can be efficiently cleaved, at the same time leaving all other protecting groups intact. Thus, a full set of all 20 trimers was synthesized by phosphotriester chemistry starting with the condensation of *N*-acyl-3'-*O*-(*o*-chlorophenylphosphate)nucleosides to 3'-*O*-(2-azidomethylbenzoyl)-protected nucelosides, followed by removal of the 5'-*O*-DMTr group and extension of the dimer to the trimer by coupling of another *N*-acyl-3'-*O*-(*o*-chlorophenylphosphate)nucleoside. The final removal of the 2-azidomethylbenzoyl group occurred by reduction of the azide with triphenylphosphine in aqueous dioxane and subsequent spontaneous intramolecular cyclization leading to cleavage of the ester bond and release of the free 3'-OH group [29] (Figure 4A).

Also with 3'-*O*-TBDMS-protected monomers as mentioned above, a full set of trimers representing codons of all 20 amino acids was synthesized, although using phosphite triester chemistry [27]. In this case, the synthesis started with the coupling of an *N*-acyl-5'-*O*-DMTr-protected nucleoside-3'-*O*-phosphoramidite to an *N*-acyl-3'-*O*-TBDMS-protected nucleoside, followed by oxidation of the internucleotide phosphorous. Upon cleavage of the 5'-*O*-DMTr group, the dimer was reacted with another *N*-acyl-5'-*O*-DMTr-protected nucleoside-3'-*O*-phosphoramidite to afford the trimer. The 3'-*O*-TBDMS group was selectively removed under mild conditions with trimethylamine/3HF (Figure 4B) with strict control of pH to leave the β-cyanoethyl groups at the internucleotide phosphates intact [27]. With both procedures (3'-*O*-(2-azidomethylbenzoyl) and 3'-*O*-TBDMS protection), 20 trinucleotides of high purity were prepared and upon phosphitylation used as synthons in oligonucleotide synthesis [27,29].

In general, the reported syntheses of trinucleotides in solution proceed by either phosphite triester chemistry or phosphotriester chemistry with the latter being the more robust method. Also H-phosphonate chemistry has been used for assembling short oligomers in solution [34], although not with the aim of generating trinucleotide synthons for gene synthesis.

### 2. Preparation of trinucleotides on solid phase

**Figure 5:**
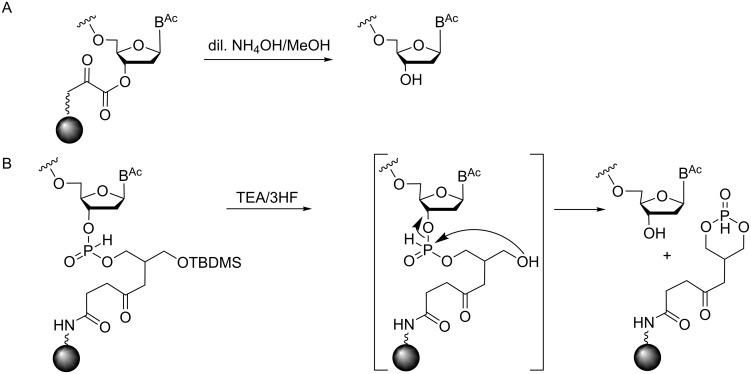
Release of trinucleotide blocks from the solid support by cleavage of an oxalyl anchor (A) and by a transesterification mechanism (B).

Given the fact that trinucleotide synthesis in solution requires tedious purification and isolation of the products after each step of the synthesis, the assembly of trimers on a solid phase appears to be an attractive alternative. However, it has to be taken into account that the 3'-start nucleoside is required to be linked to the solid phase in a way that allows the cleavage of the trimer from the solid support, but leaves all other protecting groups intact. Therefore, the routinely used succinate linkage for immobilization of the start nucleotide cannot be used. Instead, linkers that allow a release of the trimers by a non-nucleophilic and/or non-basic treatment are required. In terms of trimer synthesis only one report in the literature describes such a strategy: The start nucleoside was loaded onto controlled pore glass (CPG) via an oxalyl anchor (Figure 5A), which after the synthesis was cleaved with a 5% solution of 25% aqueous ammonia in methanol, or with 20% pyridine in methanol [20].

Combined with phosphotriester chemistry for trimer assembly, this treatment did not cause damage of the phosphotriester linkages and the nucleobase *N*-acyl groups. Using this strategy the large scale synthesis (5 g) of 3'-unprotected trinucleotides proceeded with a total 75–90% yield [20].

Other strategies with potential for the solid-phase synthesis of protected trinucleotides might rely on a universal solid support, from which oligomers with free 3'-OH function are released by a transesterification mechanism [35]. The 3'-start nucleoside is bound to one of the primary hydroxy groups of CPG-linked glycerol via an H-phosphonate linkage (Figure 5B). The removal of the TBDMS group from the remaining primary alcohol of glycerol induces the spontaneous cleavage of the H-phosphonate and the release of the oligomer with the free 3'-OH group leaving all other protecting groups intact. This strategy has been shown to be compatible with phosphoramidite chemistry and β-cyanoethyl protection of the internucleotide phosphates [33].

A more recent report describes the preparation of a polystyrene support decorated with a photolabile linker and its potential use for the synthesis of siRNA duplexes under mild and neutral conditions [36]. A similar strategy was used for the synthesis of partially 2'/3'-*O*-acetylated RNA oligonucleotides [37]. A photo-cleavable linker would also have potential for the synthesis of protected trinucleotides, as it would allow the cleavage of the trimer from the support by irradiation with UV light, without harming nucleobase and internucleotide phosphate protection. Nevertheless, photo-induced formation of byproducts may be an issue to be considered.

**Figure 6:**
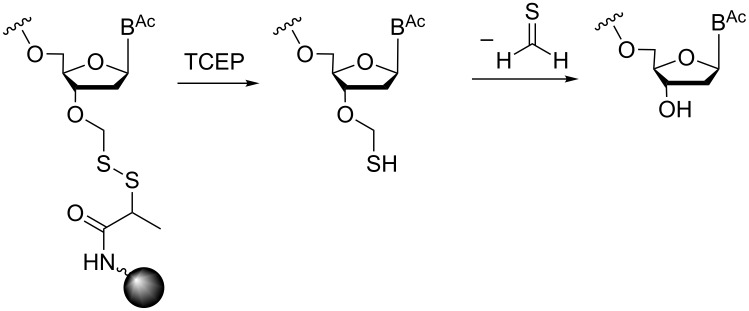
Release of the trinucleotide from the support under reductive conditions.

In our lab, we have been developing a strategy for solid-phase trinucleotide synthesis involving a disulfide linkage to the support (CPG or polystyrene), which can be cleaved under reductive conditions without harming nucleobase and phosphate protecting groups. The disulfide bridge is generated through the reaction of a 3'-*O*-methylthiomethyl-functionalized nucleoside with 2-mercaptopropionic acid and subsequent coupling to amino-functionalized CPG or polystyrene. After assembly of the trinucleotide on the support, the disulfide bridge is cleaved by treatment with dithiothreitol (DTT) [38] or tris-(2-carboxyethyl)phosphine (TCEP, Figure 6) leaving all other protecting groups intact.

The resulting hemi-(*S,O*)-acetal at the nucleotiode 3'-terminus is spontaneously degraded into the alcohol and thioformaldehyde, thus delivering the trimer with free 3'-OH group for subsequent phosphitylation. The detailed strategy and syntheses will be described elsewhere.

### 3. Preparation of trinucleotides by inverse solid-phase synthesis

Interestingly, also the use of polymer-supported reagents for H-phosphonate or phosphoramidite activation and phosphite oxidation has been described [34,39], thereby combining the advantages of solution chemistry and solid-phase methods. Thus, solid-supported acyl chloride or pyridinium tosylate as the activator of nucleoside-3'-*O*-H-phosphonates/phosphoramidites, and polystyrene-bound trimethylammonium periodate as oxidation reagent have been demonstrated to be superior for dimer and trimer synthesis, as complicated purification steps can be avoided, and excess reagents are easily removed by filtration. Compared with standard phosphotriester and phosphite triester chemistry, the limitations of this approach are lower coupling yields and side reactions hampering the yield and quality of the desired products [34,39].

### 4. Preparation of trinucleotides on soluble supports

Another strategy of combining the advantages of solution chemistry and solid-phase methods is the assembly of oligonucleotides on soluble supports. Among the supports used for this purpose, polyethylene glycol (PEG) has a prominent position, appearing as the routinely used polymer [40–44]. The isolation of intermediate and final products from the reaction mixture proceeds by precipitation from diethyl ether and filtration, thus significantly speeding up the process. In addition, the method is favorable in terms of producing oligonucleotides at a larger scale, since the reaction proceeds in homogeneous solution on a rather cheap polymer. The synthesis of oligonucleotides on soluble supports has been reviewed recently [45], showing that a variety of soluble polymers and precipitative supports are well suited to it. Also the solution-phase synthesis of protected trinucleotide building blocks has been described in the literature [21–23]. In an initial attempt, thymidine as a start nucleoside was tethered to a precipitative tetrapodal soluble support via a disulfide-linker [21] (Table 1, entry 1).

**Table 1:** Assembly of trimers on soluble supports.

entry	soluble support	5'-O-PG	chemistry	release conditions

1	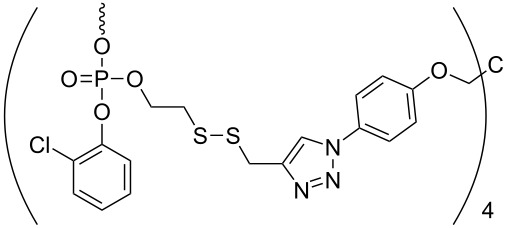	H	phosphotriester	TCEP, NEt_3_, MeOH, 3 h, 57%
2	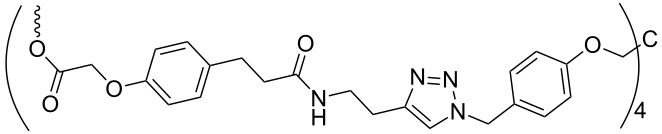	DMTr	phosphotriester	K_2_CO_3_,DCM/MeOH/ dioxane, 30 min, 88–99%
3	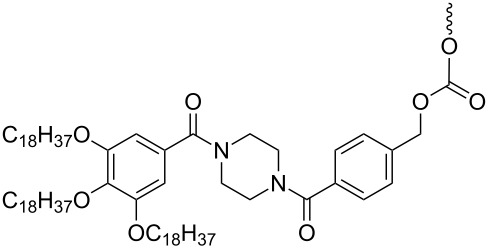	DMTr	phosphoramidite	H_2_/Pd, THF, 40 h, 44–49%

Upon detritylation, the support carrying the start nucleoside now having a free 5'-OH group was precipitated from methanol, followed by coupling with a 5'-*O*-DMTr-protected nucleoside-3'-*O*-(*o*-chlorophenyl)phosphate activated as benzotriazol and renewed precipitation with methanol. The resulting dimer was then extended to the trimer by another cycle of detritylation, precipitation, coupling and precipitation. During reductive cleavage of the disulfide bond to release the fully protected trimer from the support, unfortunately the loss of the 5'-DMTr group was observed. To overcome this hurdle, the disulfide tether was replaced in a following-up study with a Q-linker (hydroquinone-*O,O'*-diacetic acid), to be cleaved with dilute methanolic K_2_CO_3_ for the release of trimers in fully protected form. Five different trimers were assembled at 0.5 mmol scale and released form the support as described [22] (Table 1, entry 2). Thus, the fully protected trinucleotide building blocks were obtained with 65 to 70% yield from three coupling cycles, each containing two precipitations.

Yet another method for the synthesis of oligonucleotide blocks has been developed using a Cbz-type alkyl-chain-soluble support [23]. The support was attached via the benzyloxycarbonyl (Cbz) group to the 3'-OH of the starting nucleoside being adenosine, cytidine, guanosine or thymidine, and trimers were assembled by phosphoramidite chemistry (Table 1, entry 3). The support was found to disperse homogenously in the reaction solvents and to precipitate upon the addition of a polar solvent, typically methanol. After coupling of a standard phosphoramidite building block followed by oxidation with 2-butanone peroxide in dichloromethane, the resulting dimer on the support was again precipitated with methanol and filtered, before detritylation and coupling of the third monomer. The release of the trimer in fully protected form from the support was achieved by hydrogenation with Pd/C (10%) in tetrahydrofurane (THF) for 40 h at room temperature. Three fully protected trimers were prepared this way with isolated yields in the range of 44 to 49% [23].

### 5. Phosphitylation and coupling of trinucleotide synthons in solid phase DNA synthesis

**Figure 7:**
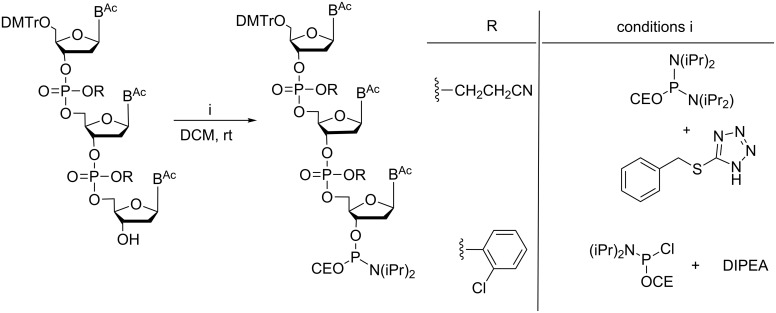
Phosphitylation of trimers. Reaction conditions, in particular the choice of the phosphitylation reagent, are dependent on the nature of the protecting group at the internucleotide phosphates.

To be used as building blocks in standard phosphoramidite synthesis, fully protected trimers need to be converted in phosphoramidites (Figure 7).

This has been described in a number of reports [19,22,27,29], and is easily achieved with trimers having *o*-chlorophenyl groups for protection of the phosphate moiety [22,29]. However, phosphitylation becomes a crucial step, if β-cyanoethyl is used as the phosphate protecting group [27]. Using 2-cyanoethyl-*N,N*-diisopropylchlorophosphoramidite for phosphitylation requires the presence of *N,N*-diisopropylethylamine (DIPEA) to neutralize HCl that is generated during the reaction. This, however, would lead to the removal of the β-cyanoethyl group at the phosphate moieties, which, due to the phosphorous atom in the oxidized state, is highly sensitive to basic agents and readily undergoes β-elimination [27].

An alternative reagent is 2-cyanoethyl-*N,N,N′,N′-*tetraisopropylphosphordiamidite in combination with tetrazole derivatives such as benzylmercaptotetrazole. Under those conditions, the phosphitylation proceeds with the production of one equivalent of diisopropylamine, which is neutralized by benzylmercaptotetrazole released back after the reaction. The tetrazole derivative is sufficiently acidic to act as a scavenger for diisopropylamine converting it into the ammonium salt. Thus, fully protected trimers can be converted to phosphoramidites without the loss of the β-cyanoethyl groups at the internucleotide phosphate linkages [27].

For the use in standard oligonucleotide synthesis, trinucleotide phosphoramidites have been dissolved in a mixture of acetonitrile and dichloromethane to a concentration of 0.1–0.15 M. The coupling yields are typically between 70–95%, preferentially with double or triple couplings, and a coupling time of 120 to 300 s [22,27,29].

## Conclusion

The synthesis of fully protected trimers can be achieved in solution, on a solid phase or on soluble supports. The key element is the choice of a suitable set of orthogonal protecting groups to allow the selective deprotection of the functionality required for the reaction, while leaving all other protecting groups intact. The first trinucleotide synthesis was performed in solution using phosphotriester or phosphoramidite chemistry. More recently strategies for trimer assembly on a solid phase or soluble supports have been developed. Here, release of the synthesized trimer in fully protected form from the support is the crucial step. This has been convincingly achieved by using molecular entities linking the trimer to the support, which can be selectively cleaved either under reductive conditions (disulfide cleavage or hydrogenation) or under mild basic conditions leaving all protecting groups at the trimer undamaged.

In particular, soluble support strategies have great potential for an efficient large scale synthesis of fully protected trinucleotides. The essential feature here is that small molecular reagents can be easily removed after coupling and 5'-*O*-deprotection, by quantitative precipitation of the soluble support in a polar solvent, such as methanol.

With the developments in the field of biotechnology and protein engineering, the preparation of gene libraries has become a major issue. In this regard, the use of trinucleotide synthons for codon-based gene synthesis has high potential, as it allows the fully controlled total or partial randomization at any predefined number and position of codons of a given gene. Methods for their large scale preparation are available now.
